# Comparative efficacy of glucocorticoid receptor agonists on Th2 cell function and attenuation by progesterone

**DOI:** 10.1186/s12865-020-00383-8

**Published:** 2020-10-19

**Authors:** Alexander Luchak, Lauren A. Solomon, Tharsan Kanagalingam, Meerah Vijeyakumaran, Brian H. Rowe, Lisa Cameron

**Affiliations:** 1 Department of Pathology and Laboratory Medicine, Western University, 1151 Richmond Street, Dental Science Building Rm. 4037, London, Ontario N6A 5C1 Canada; 2 Department of Emergency Medicine and School of Public Health, University of Alberta, Edmonton, Alberta Canada

**Keywords:** Asthma, Th2 cells, Type 2 cytokine, IL-13, Apoptosis, Corticosteroid, Sex hormone, Progesterone

## Abstract

**Background:**

Corticosteroids (CS)s suppress cytokine production and induce apoptosis of inflammatory cells. Prednisone and dexamethasone are oral CSs prescribed for treating asthma exacerbations. While prednisone is more commonly prescribed, dexamethasone is long acting and a more potent glucocorticoid receptor (GR) agonist. It can be administered as a one or two dose regime, unlike the five to seven days required for prednisone, a feature that increases compliance. We compared the relative ability of these two oral CSs to suppress type 2 inflammation. Since progesterone has affinity for the GR and women are more likely to relapse following an asthma exacerbation, we assessed its influence on CS action.

**Results:**

Dexamethasone suppressed the level of IL-5 and IL-13 mRNA within Th2 cells with ~ 10-fold higher potency than prednisolone (the active form of prednisone). Dexamethasone induced a higher proportion of apoptotic and dying cells than prednisolone, at all concentrations examined. Addition of progesterone reduced the capacity of both CS to drive cell death, though dexamethasone maintained significantly more killing activity. Progesterone blunted dexamethasone-induction of FKBP5 mRNA, indicating that the mechanism of action was by interference of the CS:GR complex.

**Conclusions:**

Dexamethasone is both more potent and effective than prednisolone in suppressing type 2 cytokine levels and mediating apoptosis. Progesterone attenuated these anti-inflammatory effects, indicating its potential influence on CS responses in vivo. Collectively, our data suggest that when oral CS is required, dexamethasone may be better able to control type 2 inflammation, eliminate Th2 cells and ultimately lead to improved long-term outcomes. Further research in asthmatics is needed.

## Background

Allergic asthma is typified by allergen-induced differentiation of T helper 2 (Th2) cells and their production of the type 2 cytokines IL-4, IL-5, and IL-13 [1]. Together these cytokines orchestrate much of the pathophysiology of asthma: IL-4 and IL-13 regulate B cell isotype switching to IgE [2] and upregulate endothelial expression of VCAM-1, mediating eosinophil infiltration [3]. IL-5 directs eosinophil differentiation and survival [4, 5] and IL-13 drives mucus production [6] and airway fibrosis [7–9]. More recently, group 2 innate lymphoid cells (ILC2) have also been shown to produce type 2 cytokines [10], though they are activated by TSLP, IL-33 or IL-25 released from the epithelium following exposure not only to allergens but also various microbes [11].

Corticosteroids (CS)s are an effective treatment for most asthmatics [12] due in part to their ability to suppress type 2 cytokine expression, both in vitro and in vivo [13–17]. While CSs also induce apoptosis of eosinophils [18] and peripheral blood mononuclear cells [19], within the T lymphocyte population there are differences in sensitivity to CSs across the various subsets. For instance, Brinkmann *et. al.* showed that memory T cells were 100-fold less sensitive to steroid-mediated reduction in clonal expansion than naïve T cells [20]. While Banuelos *et. al.* observed that Th1 cells seem more sensitive to steroid-induced apoptosis than Th17 cells [21], the susceptibility of human Th2 cells has not been well studied. Since the persistence of symptoms in difficult-to-treat asthma is largely attributed to the presence and repeated activation of long-lived memory Th2 cells and subsequent inflammation [22, 23], therapies aimed at their elimination have the potential to mediate lasting effects.

In asthma, the need for systemic (e.g., oral, intravenous or intramuscular) CS agents arise when inhaled CSs (ICS) fail to control symptoms or allergen/irritant exposure stimulates the inflammatory response and leads to an asthma exacerbation. In severe asthma, patients can be dependent on oral CS agents with symptoms worsening upon withdrawal [12, 24]. Due to well-known and serious systemic side effects [25], however, long term use of oral CS is not advised and guidelines now recommend prioritizing anti-Th2 therapies for asthma control [12, 24]. Oral CS is recommended for acute conditions, such as exacerbations of asthma, and has been shown to significantly reduce the risk of hospitalization and relapse following discharge [26]. In adults, prednisone is the most commonly prescribed oral CS [27] though an alternative is dexamethasone, a more potent agonist for the glucocorticoid receptor (GR) [27, 28]. A number of studies have compared short burst oral prednisone and dexamethasone in treating exacerbations of asthma and shown they have similar safety profiles, especially in children [29–35]. Their relative ability to suppress type 2 inflammation, however, has not been closely examined.

Here, we performed a head-to-head comparison of the relative anti-inflammatory capacity of prednisolone (the active form of prednisone) and dexamethasone in vitro*.* Sex as a biological variable is an important consideration in asthma. Women are more likely than men to develop severe asthma [36] and to relapse after an asthma attack [37]. Fluctuations in female sex hormones during the menstrual cycle [38] and pregnancy [39] are associated with worsening of asthma symptoms [40]. Since progesterone has affinity for the GR [41], this hormone may influence how CSs influence type 2 immunity. As such, we also examined the ability of progesterone to interfere with the action of prednisolone and dexamethasone. Collectively, our study provides insight into the relative efficacy of these two CS therapies and suggests their effects may be dampened in women.

## Results

### Dexamethasone more potently reduces type 2 cytokine levels than prednisolone

**Fig. 1:**
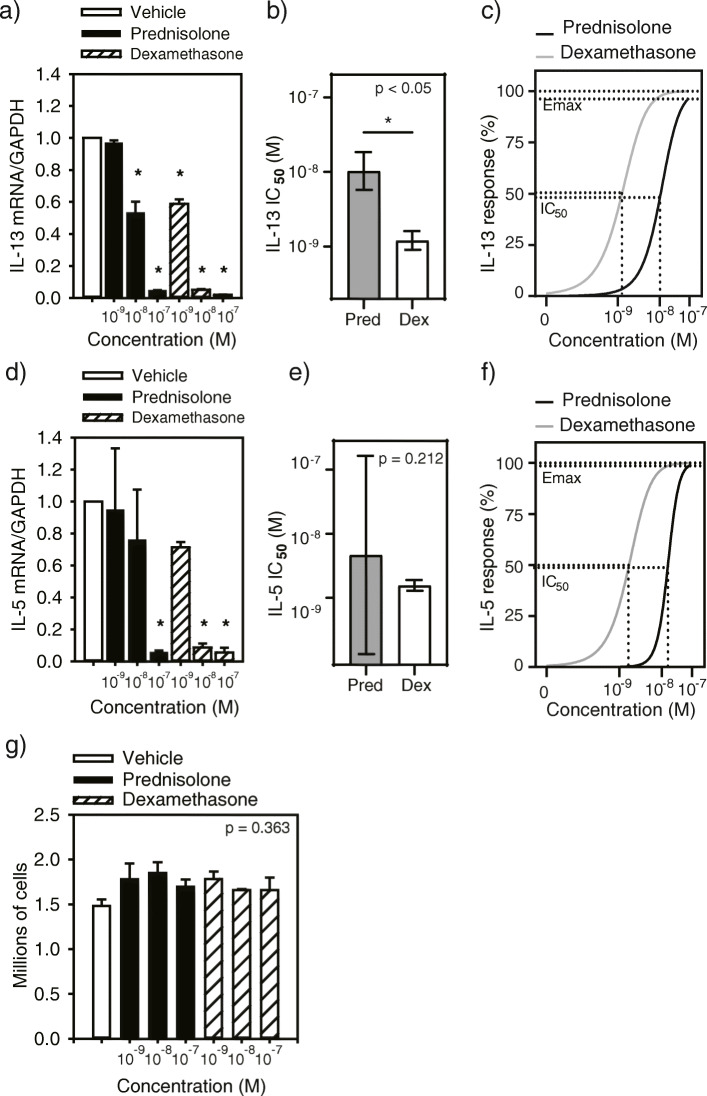
A comparison of prednisolone- and dexamethasone-mediated reduction of type-2 cytokine mRNA levels in primary Th2 cells. Fold differences in mRNA level from corticosteroid (CS) treated cells compared to vehicle are provided (*n* = 3). Quantification of *IL-13* mRNA following steroid treatment (**a**). Comparison of CS ability to suppress *IL-13* (**b**). The half maximal inhibitory concentration (IC_50_) for prednisolone and dexamethasone required to suppress *IL-13* mRNA expression (**c**). Quantification of *IL-5* mRNA following CS treatment (**d**). Comparison of the ability of either CS to suppress *IL-5* (**e**). A comparison of the IC_50_ values for prednisolone and dexamethasone required to suppress *IL-5* mRNA expression (**f**). Cell counts following culture in vehicle or increasing concentration of prednisolone or dexamethasone (**f**). Data represent mean and standard error. Pred, prednisolone; Dex, dexamethasone.*p < 0.05 determined by one-way (**a**, **d** & **g**) or two-way (**b** & **e**) RM ANOVA or t-test (**c** & **f**)

To directly compare the ability of dexamethasone and prednisolone to suppress type 2 cytokine production, primary Th2 cells (Supplemental Fig. 1 (Fig. S1)) [42, 43] cultured in IL-2 (4 days) were treated (24 h) with equimolar concentrations of either CS (10^− 9^-10^− 7^ M) as in [15, 18]. Moreover, these concentrations are within the pharmacological range [44]. IL-13 mRNA was decreased following treatment with all concentrations of dexamethasone (Fig. 1a). A 10-fold higher concentration of prednisolone was needed to significantly reduce IL-13, as the level of this cytokine was only lower in cells that had been cultured with concentrations of prednisolone ≥10^− 8^ M (Fig. 1a). The half maximal inhibitory concentration (IC_50_) of dexamethasone to suppress IL-13 mRNA was significantly lower than prednisolone (Fig. 1b), though these two CSs showed similar maximal efficacy (Fig. 1c). IL-5 mRNA level was also inhibited by both CSs, though dexamethasone was effective at a 10-fold lower concentration than prednisolone (Fig. 1d). There was no significant difference in the potency (Fig. 1e) or efficacy (Fig. 1f) of either CS to suppress IL-5 mRNA. In a broader analysis of Th2 cell response to dexamethasone we performed RNA sequencing and found that not all cytokines were suppressed; some were unchanged, some were increased and IFNγ was reduced, indicating no redirection to a Th1 phenotype (Table 1). There were also no differences in cell counts over this 24 h period, indicating that the reduction in level of cytokine mRNA was not due to cell death (Fig. 1g). These results show that dexamethasone is more potent than prednisolone in suppressing the level of type 2 cytokines.

**Table 1:** RNA-sequencing of Th2 cells

	RNA Counts		
	Vehicle	0.1 μM Dex	***P***
IL-1A	50.1 ± 17.06	34.3 ± 9.23	NS
IL-12A	14.3 ± 1.48	11.7 ± 3.01	NS
IL-15	12.9 ± 4.81	8.8 ± 1.53	NS
CD4	531.9 ± 68.93	601.1 ± 86.82	NS
IFNγ	**225.2 ± 32.86**	**79.8 ± 8.89**	**< 0.001**
IL-23A	**71.2 ± 13.80**	**95.6 ± 9.36**	**0.044**
IL-24	**5.4 ± 1.16**	**12.5 ± 4.63**	**0.029**
PGR	ND	ND	NS
PIBF1	243.5 ± 9.78	288.8 ± 27.17	NS

*Abbreviations*: *Dex* dexamethasone, *IL* interleukin, *PGR* progesterone receptor, *NS* not significant, *ND* not detected, *PIBF1* progesterone immunomodulatory binding factor 1

### Dexamethasone has a higher maximal efficacy than prednisolone to induce apoptosis

**Fig. 2:**
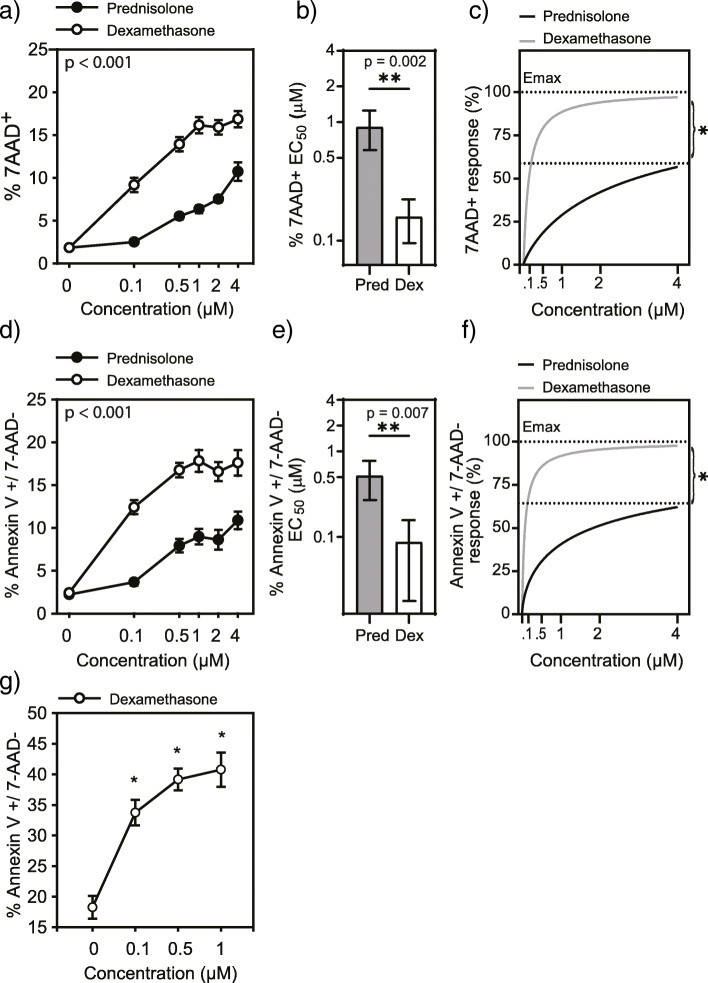
A comparison of prednisolone- and dexamethasone-induced apoptosis in a Th2 cell line (CCRF-CEM). Data are expressed as percentage of 7AAD^+^ cells (**a**) identifying dead cells. The half maximal dose (EC_50_) for prednisolone and dexamethasone required to induce necrosis of cells (**b**). Percentage of Annexin V^+^ 7AAD^−^ cells, identifying apoptotic cells (**c**). A comparison of the EC_50_ values for prednisolone and dexamethasone required to reach 50% maximal induction of apoptosis in cells (**d**; *n* = 11). Percentage of Annexin V^+^ 7AAD^−^ primary Th2 cells following treatment with dexamethasone, exhibiting a similar plateau effect as CCRF-CEM at 0.5 μM dexamethasone (**e**; n = 3). Comparison of CS-induced apoptosis in CCRF-CEM as fold-difference over vehicle (**f**). Data represent mean and standard error. Pred, prednisolone; Dex, dexamethasone. *p < 0.05 determined by two-way (**a** & **c**), *t*-test (**b** & **e**) or one-way RM ANOVA (**g**)

To assess the efficacy of dexamethasone and prednisolone to induce apoptotic cell death we used a CD4^+^ T lymphoblastoid cell line (CCRF-CEM). This line exhibits a Th2 phenotype, with enriched expression of the Th2 markers GATA3 and CRTh2 compared to Jurkat T cells (Figure Supplemental 2 (Fig. S2)). Flow cytometry was used to quantify Annexin V and 7-AAD staining following 48 h of CS treatment. Early apoptotic cells were identified as those staining for Annexin V only, but not 7-AAD, and cells positive for 7-AAD alone were considered necrotic (Fig. S3). We recently reported similar responses to dexamethasone-induced apoptosis when comparing the CCRF-CEM cell line to primary Th2 cells [45]. When we examined the response to prednisolone and dexamethasone we found that both CSs induce necrosis of CCRF-CEM cells in a dose dependent manner (Fig. 2a). Dexamethasone had a half maximal response (EC_50_) significantly lower than prednisolone (Fig. 2b) and was also the more effective inducer of cell death (Fig. 2c). Similarly, both CSs induced apoptosis (Fig. 2d), though dexamethasone was more potent (Fig. 2e) and more effective (Fig. 2f) than prednisolone. Figure 2g shows the response of primary Th2 cells to dexamethasone-induced apoptosis was similar to CCRF-CEM, reaching a plateau at 0.5 μM.

### Progesterone antagonizes corticosteroid-induced apoptosis

**Fig. 3:**
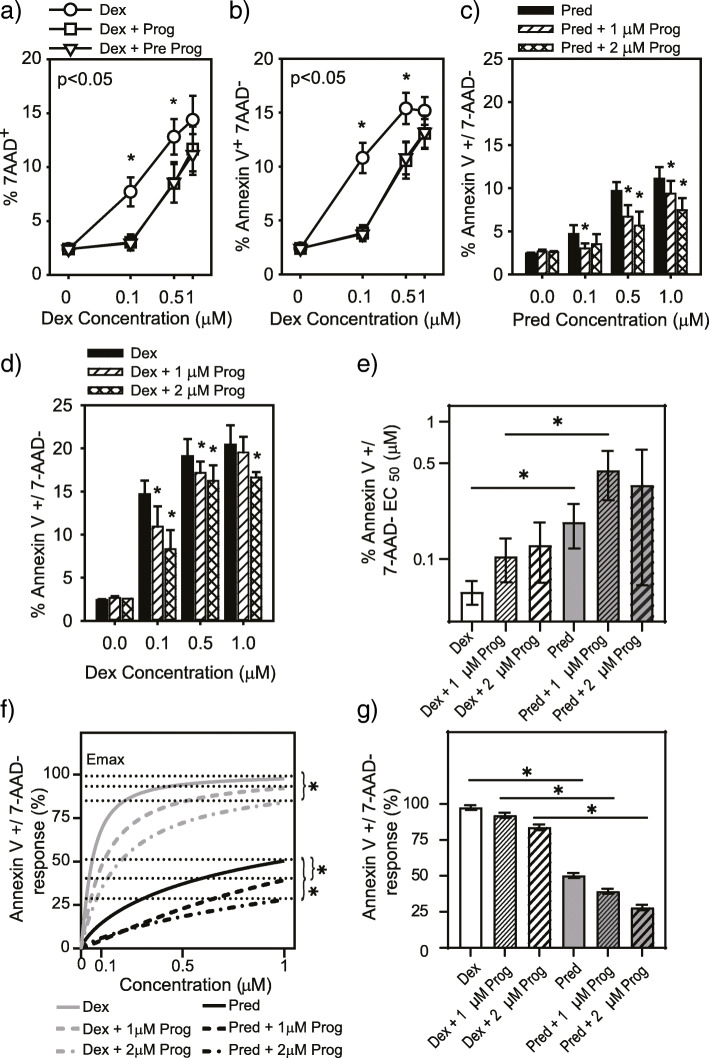
Corticosteroid-induced cell death is dampened by the female sex hormone progesterone. Dexamethasone-induced cell death (**a**, % 7AAD^+^) and apoptosis (**b**, % Annexin V^+^ 7AAD^−^) of a Th2 cell line (CCRF-CEM) in the presence or absence of progesterone (2 μM, *n* = 5). Head-to-head comparison of apoptosis following treatment with prednisolone (**c**) or dexamethasone (**d**) with or without progesterone (*n* = 4). The half maximal response (EC_50_) for dexamethasone and prednisolone in the presence of progesterone (**e**). Influence of progesterone on the maximal response of prednisolone- or dexamethasone-induced apoptosis (**f**). Efficacy of prednisolone vs dexamethasone to induce apoptosis in the presence of prednisolone (**g**). Data represent mean and standard error. Pred, prednisolone; Dex, dexamethasone; Prog, progesterone; Pre, pretreatment. *p < 0.05 determined by one-way (**a**-**e**) or two-way (**f** & **g**) RM ANOVA

Since progesterone has affinity for the GR [41], we investigated whether this sex hormone influences the efficacy of CS action. Th2 cells (CCRF-CEM) were incubated with dexamethasone for 48 h in the presence or absence of progesterone and cell death and apoptosis assessed by flow cytometry. We found that progesterone, in the absence of dexamethasone, had no effect but when added with or 30 min prior to dexamethasone significantly reduced both cell death (Fig. 3a) and apoptosis (Fig. 3b). To assess the differential effect of progesterone on either CS, Th2 cells were cultured with prednisolone or dexamethasone in the presence or absence of progesterone. Head-to-head comparison revealed that the proportion of apoptotic cells induced by prednisolone (Fig. 3c) and dexamethasone (Fig. 3d) was reduced by progesterone, though no significant change in EC_50_ for either CS was observed (Fig. 3e). Progesterone did reduce the maximal efficacy of both CSs (Fig. 3f), though dexamethasone maintained superior killing activity over prednisolone at each progesterone concentration (Fig. 3g).

### Progesterone inhibits GR signaling

**Fig. 4:**
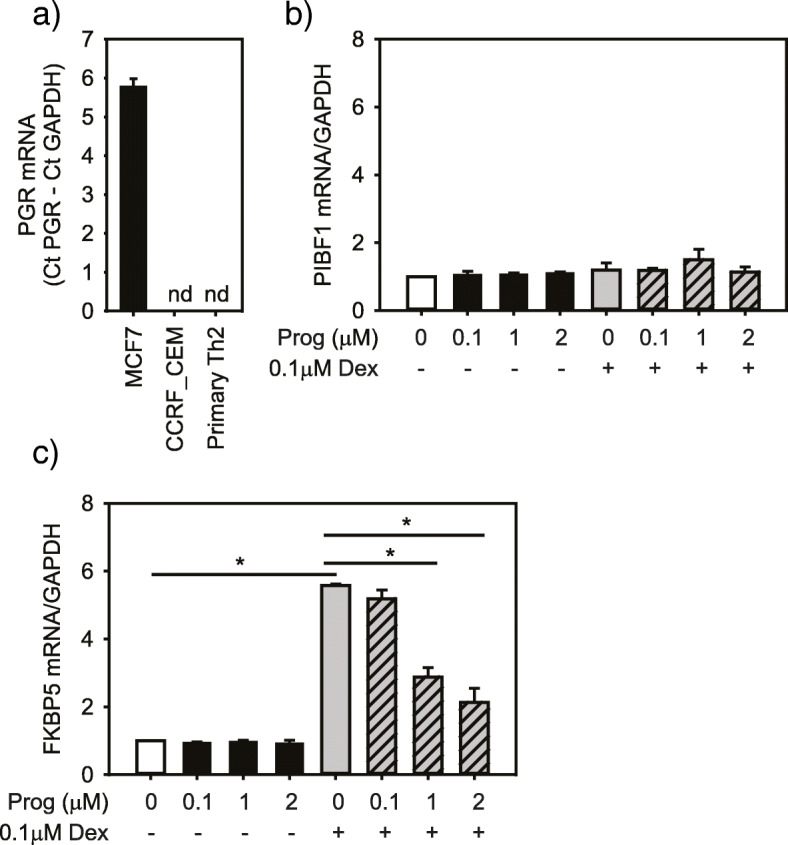
Expression of the nuclear progesterone receptor (PGR) is undetectable in a Th2 cell line (CCRF-CEM) and primary Th2 cells, but present in a breast adenocarcinoma cell line (MCF-7) used as a positive control (n = 3; **a**). Effect of progesterone with or without dexamethasone on expression of *PIBF1* (n = 3, **b**)*.* Progesterone reduced the dexamethasone-mediated increase in *FKPB5* mRNA level, but had no effect when applied alone (n = 5, **c**). Data represent mean and standard error. *p < 0.05 determined by one-way ANOVA

To investigate the mechanism by which progesterone reduced the CS effect, we examined expression of the nuclear progesterone receptor (*PGR*). We found that in the Th2 cell line (CCRF-CEM) and primary Th2 cells *PGR* mRNA was undetectable by qRT-PCR, though present in a breast cancer cell line (MCF-7) (Fig. 4a). We also found no induction of progesterone immunomodulatory binding factor 1 (PIBF1), a factor known to be induced by progesterone [46, 47] (Fig. 4b), in CCRF-CEM nor primary Th2 cells treated with dexamethasone (Table 1). These results suggest that the progesterone effect on CS-induced apoptosis was not through progesterone receptor signaling. Since progesterone has also been shown to have affinity for the GR [41], we next assessed whether it could influence the dexamethasone mediated induction of FKBP5 mRNA*,* a gene highly induced by CS [45, 48, 49]. Indeed, the level of FKBP5 mRNA following exposure to dexamethasone was significantly blunted when progesterone was added to the culture (Fig. 4c), indicating this sex hormone interferes with GC:GR signaling.

## Discussion

Prednisone and dexamethasone are both oral CS therapies recommended for treating patients with moderate to severe asthma exacerbations. While prednisone is more commonly used [27], studies in children and adults have shown that dexamethasone has a similar safety profile and efficacy in terms of reducing relapse rates, hospitalizations, symptoms and time to return to normal activity [29, 30, 33–35]. Moreover, its long half-life and shorter treatment duration have proven popular among patients, caregivers and clinicians, especially in children with acute asthma. Dexamethasone is a more potent GR agonist than prednisone [28]; however, whether it is more effective in reducing inflammation is less well understood. Here, we examined the anti-inflammatory effects of these two CS agents in vitro and found that dexamethasone was both more potent and more effective than prednisolone. As such, our data suggest that treating exacerbating asthmatics with dexamethasone may result in better control of symptoms and/or severity of disease, aspects of asthma mediated by persistent inflammation.

Studies comparing the effectiveness of standard dose regimes of dexamethasone (12–16 mg/day, 1–2 days) to prednisone (60 mg/day, 5 days) found no difference in asthma relapse rates within two weeks [33–35]. These studies, however, failed to examine longer time points or differential CS effects on cellular or cytokine responses. Our study shows that dexamethasone significantly decreased IL-13 mRNA at a 10-fold lower concentration (10^− 9^ μM) than prednisolone. IL-5 appeared to be generally less CS sensitive, with significant suppression observed only at 10^− 8^ μM dexamethasone. However, at the highest concentration examined (10^− 7^ μM), still physiologically relevant [44], both CS agents almost completely suppressed IL-13 and IL-5 mRNA levels. This result indicates that in vivo type 2 cytokines are likely sufficiently controlled with an optimal dose of either prednisolone or dexamethasone and, in line with other reports, are anticipated to reduce the proportion of CD4^+^ T cells expressing IL-4 and IL-5 [50]. However, we recently showed that the suppressive effect of dexamethasone on IL-13 was reversed after activation unless cells were continuously exposed to dexamethasone [45], suggesting the effect could be temporary. The lack of increase in IL-10 or IL-17 following dexamethasone treatment [45], coupled with our new data showing that dexamethasone reduced IFNγ mRNA levels (Table 1), indicates active suppression of type 2 cytokine transcription, rather than shifting Th2 cells toward a Th1, Th17 or T regulatory cell phenotype.

Type 2 cytokines mediate many features of asthma and therapies blocking their action are effective and are now recommended as frontline controllers in severe asthma [24]. Attention, however, is now turning toward therapies that reduce the proportion and/or development of type 2 cytokine-producing cells with the long term hope of actually modifying or curing asthma [51]. Circulating Th2 cells are an important aspect of immune memory and their level controls a patient’s susceptibility to respond to allergens and the development of persistent symptoms. For this reason, we examined differences in the ability of these two CS agents to induce Th2 cell apoptosis. We found that dexamethasone was superior to prednisolone in driving apoptosis (~ 10-fold more effective) - no matter the concentration added, prednisolone was never able to induce apoptosis to a similar degree as dexamethasone. These results suggest prednisolone is a partial agonist, relative to dexamethasone, similar to previous studies showing this in terms of gene expression [52] and the ability of dexamethasone to enhance prednisolone-induced cytotoxicity [53].

Prednisolone failed to match the degree of dexamethasone-induced apoptosis, but was equi-effective in cytokine suppression. This difference may be due to mechanism(s) of action. Corticosteroid suppression of IL-5 and IL-13 has been shown to be due to GR interfering with transcription factors binding to the promoter of these type 2 cytokine genes [14], while GC-induced apoptosis has been associated with induction of pro-apoptotic genes such as BIM (BCL-2-interacting mediator of cell death) [54]. Our results suggest that dexamethasone more effectively induces pro-apoptosis genes than prednisolone. Indeed, others have shown differential effects of various steroids on gene expression; in airway cells dexamethasone, fluticasone, ciclesonide (and others) were equally effective in driving the expression of some genes (GILZ, p57^kip2^), but not others (PDK4) [55].

Clearly, studies are needed to examine the effect of dexamethasone compared to prednisone in patients experiencing asthma exacerbation, but this is challenging since dexamethasone is more often prescribed to children and prednisolone is the oral CS of choice in adults. A few studies have compared these two CSs and found they were associated with similar rates of relapse and hospitalization within 10–14 days [34, 35], providing little rationale to recommend a change in prescribing habits. Those studies, however, did not assess whether dexamethasone had a stronger suppressive effect on immune cell responses such as the degree of Th2 cell apoptosis. There is evidence to suggest that stronger GR agonists provide better outcomes; Demirca *et. al.* showed type 2 cytokine expression at day 7 following ex vivo activation of peripheral blood mononuclear cells was higher in patients who received oral methylprednisolone post-exacerbation compared to those who received the more potent fluticasone propionate [56]. Our mechanistic data suggest that the higher potency dexamethasone induced more Th2 cell apoptosis than the weaker agonist prednisone. In vivo, dexamethasone may be better able to eliminate Th2 cells thereby reducing persistent symptoms driven by type 2 inflammation and could result in fewer sub-acute relapses and/or repeat exacerbations. As such, our in vitro data beseech clinical centers to perform head-to-head comparisons of these two CS agents to determine their relative in vivo effect on type 2 inflammation and persistence of symptoms.

Since corticosteroid withdrawal studies have shown that suppression of type 2 inflammation requires continuous exposure to steroid [45, 57, 58], therapeutic approaches aimed at eliminating Th2 cells may provide more sustained repression of allergic responses. Th2 cells are highly differentiated with a strong ability to survive, mediated through expression of the anti-apoptotic factor BCL-2 [59, 60]. The BCL-2 inhibitor ABT-199, in clinical trials for leukemia [61], was also shown to reduce the level of airway eosinophils and Th2 cells in a mouse model of asthma [62]. Huang *et. al.* showed that elimination of Th2 cells (using an antibody against CRTh2) resulted in significantly fewer eosinophils and lower levels of type 2 cytokines and chemokines in the blood, lymph nodes and lung in a mouse model of asthma [63]. Ultimately, therapies targeting development of Th2 cells, such as those blocking the IL-4Rα or the cytokine TSLP [64, 65], may prove the most effective in reducing persistence of asthma symptoms.

Women and men respond differently to acute asthma. For example, women are more likely to relapse following an asthma exacerbation than men [37]. Furthermore, the female sex hormone progesterone has affinity for the GR [41]. As such, we examined whether progesterone could dampen the ability of CS to induce Th2 cell apoptosis and its mechanism of action. We found that progesterone (1 μM, considered to be a physiologic level during pregnancy [41]), interfered with both prednisolone and dexamethasone and that the effect was identical if hormone was added in combination or as a pre-treatment. The Th2 cell line (CCRF-CEM) and primary Th2 cells had no PGR mRNA and no production of PIBF1 mRNA in response to progesterone, indicating that the effect was not due to activation of nuclear progesterone receptors [46]. We did, however, find that progesterone reduced the level of FKBP5 mRNA, a gene known to be induced by GC exposure and GR binding to its promoter [48, 49]. As such, our data suggest that progesterone antagonizes GC:GR signaling, rather than acting through its own receptor. This finding is in line with Guo *et. al.*, who showed that treating murine NK cells with the progesterone analog P4, in the presence of PGR blockade, antagonized GR signaling and reduced IFNγ and CD69 expression following CpG/IL-12 stimulation [66]. Progesterone was also shown to antagonize dexamethasone-induced apoptosis of murine thymocytes [67]. Collectively these studies support our hypothesis that circulating progesterone levels may influence the efficacy of CS action in vivo, which could explain why some women experience worse symptoms at times when progesterone levels are elevated [38, 39] and are more likely to be diagnosed with severe asthma [36].

Dexamethasone is a long-acting CS (36–72 h half-life) with 30 times more GR activity than hydrocortisone. Due to these factors, long term dexamethasone treatment is associated with serious side effects, such as suppression of the hypothalamic-pituitary-adrenal (HPA) axis, so is generally reserved for treating acute symptoms like exacerbations of asthma [27]. Prednisolone is less potent (4 times the activity of hydrocortisone) and is shorter acting (12–36 h half-life) [27], so historically it has been used as a controller medication. In 2012, due to the known side effects of long term oral steroid use, the GINA guidelines downgraded the use of oral CS in chronic asthma and recommend them only if other controllers, such as anti-IgE and anti-IL-5, were not available or did not work [68]. Nevertheless, a recent study of severe asthmatics reported a third of their study population was using oral prednisone, with an average duration of 4 years and mean dose 17.5 mg/day [69]. The ranges of oral prednisone prescribed vary greatly from very low (1–7.5 mg/day) to higher doses (10–40 mg/day) [68]. Though less than recommended for exacerbation (60 mg/day) [34, 35], these doses equate to in vitro concentrations of ~ 0.3–0.8 μM (7.5–17.5 mg/day). In light of our data, the dose range of prednisolone for chronic use may be sufficient to suppress type 2 cytokine levels, but relatively inefficient as an inducer of apoptosis, particularly in women. These data indicate that inhaled CS formulations of higher affinity GR agonists, such as fluticasone furoate [70], may also be effective in eliminating Th2 cells and suggest this should be examined in future studies.

## Conclusion

Our study provides an in vitro examination of the anti-inflammatory capacity of two clinically relevant corticosteroids, prednisolone and dexamethasone. We found that dexamethasone more potently and effectively reduced type 2 cytokine expression and diminished Th2 cell numbers than prednisolone. In the presence of progesterone, dexamethasone maintained a superior ability to drive Th2 cell death over prednisolone. Though further study is needed to assess these effects in patients with asthma, our results do suggest that amongst oral CSs dexamethasone may be the better therapeutic option for treating exacerbations of asthma, particularly in women.

## Methods

This study was approved by the Western University Health Sciences Research Ethics Board (Approval number 106770). All subjects gave informed consent.

### Th2 cells

Primary Th2 cells were differentiated from CD4^+^ T cells obtained from healthy donor peripheral blood mononuclear cells (PBMCs) and cultured under Th2 differentiating conditions as previously described [43]. Th2 cells were maintained on cycles of IL-2 and plate bound αCD3/αCD28 (3 days) or just IL-2 (4 days) at 2 × 10^6^ cells/mL. The immortalized cell lines CCRF-CEM (ATCC® CCL-119™) and Jurkat (ATCC® E6–1, TIB-152™) were purchased from American Type Culture Collection (VA). They are both CD4^+^ T cell lines derived from acute human T cell leukemia. Cell lines were grown in RPMI-1640 media (Sigma-Aldrich) supplemented with 10% FBS (Hyclone Scientific, Fisher Scientific, ON, Canada) and 1X penicillin-streptomycin-glutamine (Gibco, Invitrogen, Thermo Fisher Scientific). Cells were incubated at 37 °C, in 85% humidity and 5% CO_2_ and maintained at 0.2–0.3 × 10^6^ cells/mL with re-seeding every 2 days.

### Cell culture conditions

Dexamethasone, prednisolone and progesterone (Sigma Aldrich, ON, Canada) were prepared in 100% ethanol and serially diluted in culture medium prior to treatment of primary Th2 cells (1.3 × 10^6^ cells/mL) and CCRF-CEM cells (0.2 × 10^6^ cells/mL). While prednisone is most commonly prescribed to patients, it needs to be metabolized in the liver to prednisolone, the active drug. Therefore, we used prednisolone for these in vitro experiments. Cells were treated with 10^− 9^ – 10^− 7^ μM of each CS agent as in [15, 18]. These concentrations were based on the relative anti-inflammatory activity of dexamethasone being 6.25-fold more potent than prednisolone (https://clincalc.com). Moreover, they are within the pharmacological range - oral administration of dexamethasone (12 mg, ~ 0.26 μM) and prednisone (60 mg, ~ 2.76 μM) [44]. For experiments with progesterone, cells were either incubated with progesterone for 30 min prior to dexamethasone or added simultaneously with dexamethasone. Gene expression was assessed after 24 h and staining for flow cytometry conducted after 48 h.

### Gene expression

#### Quantitative

*RT-PCR* RNA (400 ng) was isolated with RNeasy Plus Mini/QIAshredder (Qiagen, Hilden, Germany) and reverse transcribed using iScript Reverse Transciption Supermix (Bio-Rad, Hercules, California). Real time polymerase chain reaction (RT-PCR) was conducted using TaqMan Fast Advanced Master Mix (Applied Biosystems, Foster City, California). Assays for IL-13 (Hs00174379_m1), IL-5 (Hs01548712_g1), PGR (Hs01556702_m1), PIBF1 (Hs00197131_m1), FKBP5 (Hs01561006_m1), CRTh2 (Hs00173717_m1), GATA3 (Hs00231122_m1), IFNγ (Hs00989291_m1) and the housekeeping gene GAPDH (Hs02786624_g1) were used (ThermoFisher). Thermal cycling was performed according to manufacturer’s instruction. Fold increase relative to the control condition was assessed for experimental treatments using the 2^-ΔΔ*CT*^ *method.*

#### RNA-sequencing

Messenger RNA (mRNA) sequencing was previously conducted on primary Th2 cells treated for 24 h in vehicle or 0.1 μM dexamethasone as described [45]. Normalized expression counts are presented from samples treated with ethanol (vehicle) or dexamethasone at 0.1 μM for 24 h.

### Flow Cytometry

#### Cytokine expression

Intracellular staining to characterize primary Th2 cells was previously described in [42, 43]. Briefly, cytokine staining for IL-4, IL-13 and IFNγ was performed following 4 h of stimulation with PMA (20 ng/mL) and ionomycin (1 μM) in the presence of Brefeldin A (10 μg/mL, all from Sigma Aldrich, Canada). Cells were fixed on ice (10 min) with paraformaldehyde (4%; Sigma Aldrich, Canada) and permeabilized on ice (10 min) with saponin (0.4%, Sigma Aldrich, Canada). Cells were incubated with at 4 °C (30 min) with IL-13-PE (Clone JES10-5A2, isotype rat IgG1κ PE), IFNγ-Alexa 647 (Clone B27, isotype mouse IgG1κ Alexa 647) or IL-4-Alexa 488 (Clone 8D4–8, isotype mouse IgG1κ Alexa 488) antibodies (BD Pharmingen, ON, Canada) and then washed with buffer. Fluorescence was assessed immediately using a FACS Calibur (Becton Dickson, ON, Canada) and data analyzed using FlowJo (Tree Star Inc., Ashland, OR, USA).

#### Apoptosis

Following 48 h CS treatment, CCRF-CEM or primary Th2 cells (0.5 × 10^6^ cells/ml) were washed with FACS buffer (0.5% bovine serum albumin, 0.1% sodium azide, 3% FBS), pelleted at 4 °C, re-suspended in Annexin V binding buffer (100 μL/condition; 10 mM HEPES pH 7.4, 140 mM NaCl, 2.5 mM CaCl_2_) and stained with Alexa Fluor 647 Annexin V (1 μL) and 7-AAD Viability Staining Solution (5 μL) (BioLegend, CA, USA) for 15 min. Cells were diluted with additional Annexin V binding buffer (400 μL) and data acquired using an LSR II (BD Biosciences). Flow cytometry analysis was conducted using FlowJo (Version 10, Ashland, OR, USA) and reported as the proportion of total cell population.

### Statistical analysis

Two- and one-way repeated measures analysis of variance (ANOVA) were used to determine statistical significance at *p* <  0.05 (SigmaPlot, version 12.5).

## Supplementary information

**Additional file 1 Supplemental Fig. S1.** Representative flow cytometry of primary Th2 cells differentiated from human CD4^+^ T cells. Gating strategy used to determine percentage of cells positive for IL-4 or IL-13 (vs isotype controls) and to demonstrate a polarized type 2 cytokine profile (IL-13 vs IFNγ). **Supplemental Fig. S2.** The CCRF-CEM cell line exhibits a Th2 phenotype The CD4^+^ T lymphoblastic leukemia cell line CCRF-CEM are enriched in mRNA levels of CRTh2 and GATA3, markers of the Th2 cell phenotype, compared to Jurkat, another CD4^+^ T cell line (*n* = 3, A). Type 2 (IL-13) and type 1 (IFNγ) cytokine levels in CCRF-CEM and Jurkat cells following stimulation (24 h) with PMA (20 ng/ml) and ionomycin (1 μM) (B). Data represent mean and standard error. *p <  0.05 determined by one-way ANOVA. **Supplemental Fig. S3.** Gating strategy used to determine the percentage of Annexin V^+^ and 7-AAD^−^ positive cells.

## Data Availability

The datasets used and/or analyzed during the current study are available from the corresponding author on reasonable request.

## Abbreviations

CS: Corticosteroid
GR: Glucocorticoid receptor
GC: Glucocorticoid
IL: Interleukin
Th: T helper
Ig: Immunoglobulin
ICS: Inhaled corticosteroid
FKBP5: FK506 binding protein 5
VCAM-1: Vascular cell adhesion protein 1
TSLP: Thymic stromal lymphopoietin
mRNA: Messenger ribonucleic acid
IC_50_: Half maximal inhibitory concentration
EC_50_: Half maximal effective concentration
GATA3: GATA binding protein 3
CRTh2: Chemoattractant receptor homologous molecule expressed on Th2
7-AAD: 7-aminoacitomycin D
PGR: Nuclear progesterone receptor
qRT-PCR: Quantitative real-time polymerase chain reaction
IFN: Interferon
PIBF-1: Progesterone immunomodulatory binding factor 1
BIM: BCL-2-like protein 11
BCL-2: B-cell lymphoma 2
GILZ: Glucocorticoid-induced leucine zipper
p57^kip2^: Cyclin-dependent kinase inhibitor 1C
PDK4: Pyruvate dehydrogenase kinase 4
NK: Natural killer
HPA: Hypothalamic-pituitary-adrenal
GINA: Global Initiative for Asthma
PBMC: Peripheral blood mononuclear cell
FBS: Fetal bovine serum
GAPDH: Glyceraldehyde 3-phosphate dehydrogenase
PMA: Phorbol myristate acetate
PE: Phycoerythrin
FACS: Fluorescence-activated cell sortig
HEPES: 4-(2-hydroxyethyl)-1-piperazineethanesulfonic acid
RM ANOVA: Repeated measures analysis of variance
