# Supplementary material for: Comparative efficacy of glucocorticoid receptor agonists on Th2 cell function and attenuation by progesterone
Source: BMC Immunol. 2020 Oct 19;21:54. doi: 10.1186/s12865-020-00383-8 (PMC7574173; doi:10.1186/s12865-020-00383-8)

Supplemental Figure 1

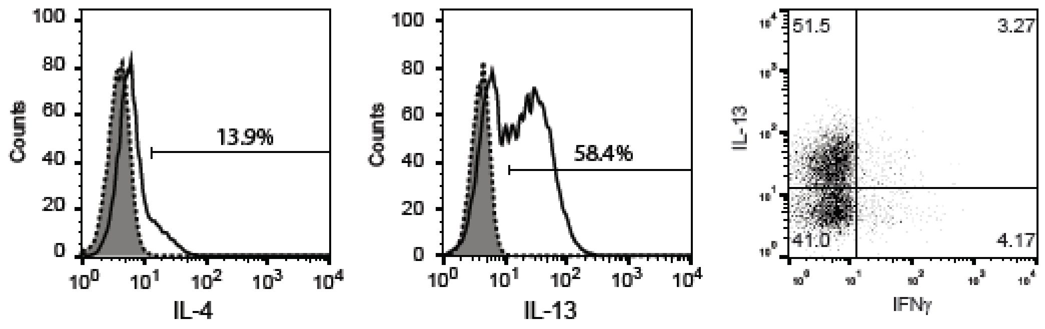

Supplemental Figure 2

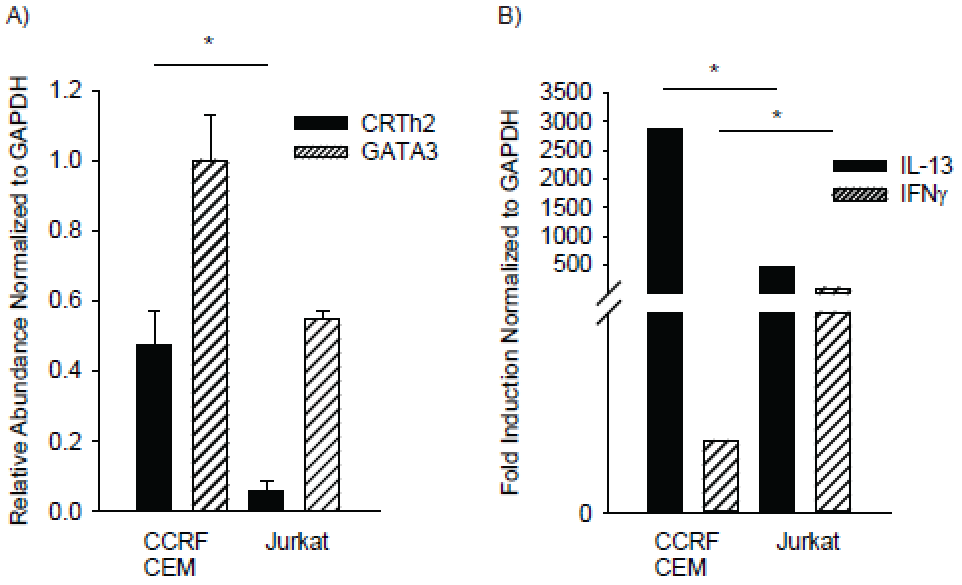

Supplemental Figure 3

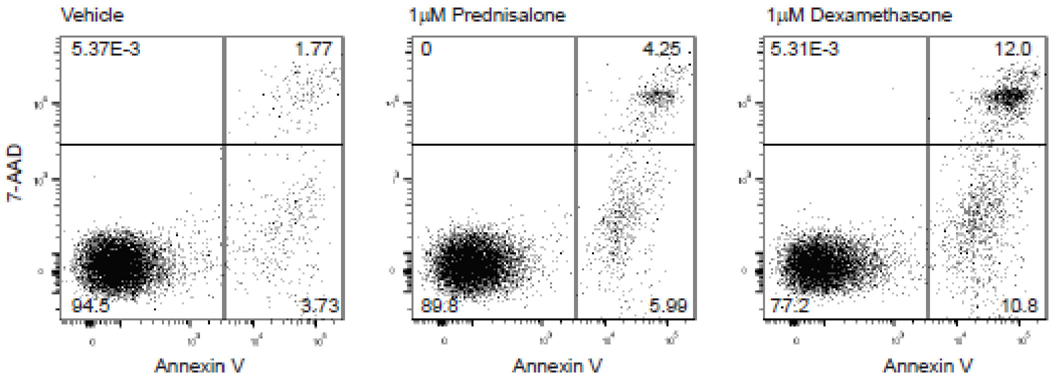

Supplemental Figure 3

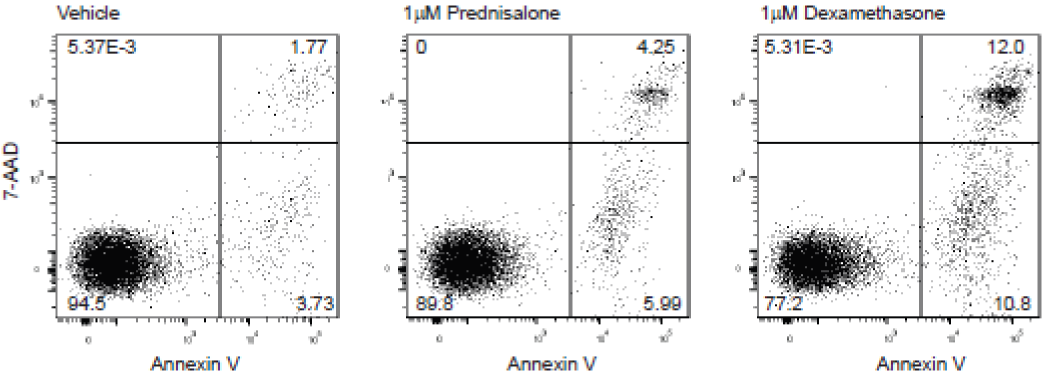

Supplement: Supplementary file 1 [file 12865_2020_383_MOESM1_ESM.docx]
